# Patterns of Ecological Adaptation of *Aedes aegypti* and *Aedes albopictus* and *Stegomyia* Indices Highlight the Potential Risk of Arbovirus Transmission in Yaoundé, the Capital City of Cameroon

**DOI:** 10.3390/pathogens9060491

**Published:** 2020-06-20

**Authors:** Armel N. Tedjou, Basile Kamgang, Aurélie P. Yougang, Theodel A. Wilson-Bahun, Flobert Njiokou, Charles S. Wondji

**Affiliations:** 1 Department of Medical Entomology, Centre for Research in Infectious Diseases, 15391 Yaoundé, Cameroon; aurelie.yougang@crid-cam.net (A.P.Y.); wilsontheodel9@gmail.com (T.A.W.-B.); charles.wondji@lstmed.ac.uk (C.S.W.); 2 Department of Animal Biology and Physiology, Faculty of Sciences, University of Yaoundé I, 812 Yaoundé, Cameroon; njiokouf@yahoo.com; 3 Department of Animal Biology and Physiology, Faculty of Sciences and Technology, Marien Ngouabi University, Brazzaville, Congo; 4 Vector Biology Department, Liverpool School of Tropical Medicine, Pembroke Place, Liverpool L3 5QA, UK

**Keywords:** *Aedes albopictus*, *Aedes aegypti*, *Stegomyia* indices, arbovirus, Cameroon

## Abstract

The dynamic of arbovirus vectors such as *Aedes aegypti* and *Ae. albopictus* remains poorly understood in large cities in central Africa. Here, we compared the larval ecology, geographical distribution and degree of infestation of *Ae. aegypti* and *Ae. albopictus* in Yaoundé, the capital city of Cameroon, and estimated their *Stegomyia* indices revealing a significant potential risk of arbovirus transmission. An entomological survey was conducted in April–May 2018 in a cluster of houses randomly selected. Each selected house was inspected, the number of inhabitants was recorded, and potential and positive containers for *Aedes* were characterized. *Stegomyia* and pupae-based indices were estimated. Overall, 447 houses and 954 containers were inspected comprising 10,801 immature stages of *Aedes* with 84.95% of *Ae. albopictus* and 15.05% of *Ae. aegypti*. Both species bred mainly in discarded tanks and used tyres, associated with turbid water and the presence of plant debris inside containers. *Aedes albopictus* was the most prevalent species in almost all neighbourhoods. The house index, Breteau index, and container index were higher for *Ae. albopictus* (38.26%, 71.81%, and 29.61%) compared to those of *Ae. aegypti* (25.73%, 40.93%, and 16.88%). These indices are high compared to the thresholds established by Pan American Health Organization and World Health Organization, which suggests a high potential risk of arbovirus transmission.

## 1. Introduction

*Aedes*-borne diseases such as dengue, yellow fever, Zika and chikungunya have emerged in several tropical and subtropical regions worldwide [1]. The viruses that cause these diseases are transmitted to humans by a bite from infected mosquitoes belonging to the *Aedes* genus, notably *Aedes aegypti* (Linnaeus 1762) and *Aedes albopictus* (Skuse 1894) [2].

*Aedes aegypti*, native to the African continent [3,4], has a cosmo-tropical distribution while *Ae. albopictus*, originating from the south-east Asian forest, has invaded all five continents over the last four decades [5,6,7,8]. The rapid spread of *Ae. albopictus* across the world may be due to the international commercialization of used tyres [9] and its strong ecological plasticity which allows it to adapt to different environments [10]. *Aedes albopictus* was reported for the first time in central Africa in Cameroon in the early 2000s [11] and has progressively colonized almost all countries in the region [12]. Despite the fact that both species have a different origin, these mosquitoes have a similar ecology, ovipositing in man-made water containers [13,14] and feeding generally on human blood [15,16]. In fact, the coexistence of both species has been well documented [14,17]. In the sympatric areas notably in Africa, both species have often colonized the same breeding sites with the difference that *Ae. albopictus* breeds preferentially in containers surrounded by the presence of vegetation while *Ae. aegypti* prefers man-made containers located in neighbourhoods with high building density [18,19,20]. It has been demonstrated that the coexistence of both species is due to the segregation of the habitat according to macro-environmental factors such as building density and vegetation index [14,18,19,21].

The competitive displacement of *Ae. aegypti* after the invasion of *Ae. albopictus* has been documented in Brazil [14,22,23], Florida [14,24], and Australia [25,26] and was suspected in several countries and territories such as La Reunion [27,28], Mayotte [29], the Republic of the Congo [30] and the Central African Republic [19]. In contrast, in Asia, *Ae. aegypti* has an overall competitive advantage over *Ae. albopictus*, especially in urban areas [31,32]. Previous studies in Cameroon demonstrated that *Ae. aegypti* is found across the country whereas *Ae. albopictus* has a distribution limited in the southern part of the country and tends to replace the resident species *Ae. aegypti* [33,34]. Detailed studies conducted in Yaoundé (Cameroon) in 2007 globally highlighted the predominance of *Ae. albopictus* but showed no significant difference between the geographical distribution of the two species according to the environment [18]. As both *Aedes* species share the same ecological niche and probably exploit the same resources in Yaoundé, it was suggested that the competition between them is ongoing but no species had yet taken over, and a decade later there is a need to assess the state of this competition. In addition, the current *Stegomyia* indices of both species remain unclear or need to be updated to adjust vector control strategies in order to prevent arbovirus outbreaks.

*Aedes*-borne diseases such as dengue especially were once considered scarce in Cameroon. However, during the past decade, cases of dengue have increasingly been reported [35,36,37,38,39] suggesting an active circulation of this virus in Cameroon. In addition, vector competence analysis showed that both *Ae. albopictus* and *Ae. aegypti* from central Africa are able to transmit the yellow fever virus [40], dengue virus [41], and Zika virus [42]. Until now, apart from yellow fever for which there is a vaccine, there has been no specific treatment or effective vaccine against these viruses. Thus, vector control and surveillance remain the cornerstone of preventing *Aedes*-borne diseases. In this study we present comparative data on the current geographical distribution, larval ecology, and level of infestation of *Ae. aegypti* and *Ae. albopictus* in Yaoundé based on the *Stegomyia* indices.

## 2. Results

### 2.1. Pre-Imaginal Infestation

Overall, 447 houses in 30 neighbourhoods were investigated with 4471 inhabitants and an average of ten persons per house. A total of 955 potential containers were prospected from which 360 (37.7%) were found to be positive for the aquatic stages of *Ae. aegypti* and/or *Ae. albopictus.* Several other mosquito species were identified in association with *Ae. albopictus* and/or *Ae. aegypti* during the survey, including *Ae. simpsoni* (Theobald 1905), *Anopheles gambiae* s.l. (Giles 1902), *Culex tigripes* (De Grandpré and De Charmoy 1900), *Culex pipiens quinquefasciatus* (Say 1823), *Culex antennatus* (Beker 1903), *Culex* sp. (Linnaeus 1758), *Eretmapodites brevipalpis* (Ingram and De Meillon 1927), and *Toxhorynchites brevipalpis* (Ribeiro 1991). Overall, the proportion of containers infested by *Ae. albopictus* was significantly higher than that infested by *Ae. aegypti* (*χ*^2^ = 37.78, *df* = 1, *p* < 10^−9^). Similarly, the proportion of containers infested by *Ae. albopictus* (28.1%) was significantly higher than that infested by *Ae. aegypti* (15%) in downtown as well as in suburban (*Ae. albopictus*: 44.7%; *Ae. aegypti*: 18.6%) areas (*p* < 0.01), whereas no significant difference was observed in rural areas (*p* > 0.05) (Table S1). The proportion of containers infested by *Ae. albopictus* only (49.2%), *Ae. aegypti* only (10.8%) and by both species (40%) differed significantly (*χ*^2^ = 34.1, *df* = 2, *p* < 10^−8^) (Table 1). Of the 30 neighbourhoods, only one was negative for *Aedes* species, the Briqueterie neighbourhood where no immature stage of *Ae. albopictus* and/or *Ae. aegypti* was reported in houses surveyed (Table 1, Table S1).

### 2.2. Larval/Pupal Indices and Risk of Dengue and Yellow Fever Transmission

**Figure 1:**
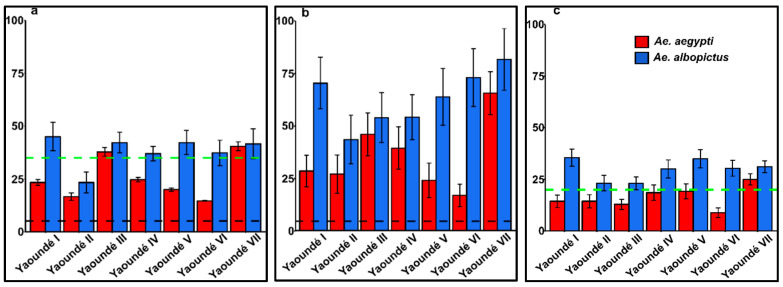
*Stegomyia* indices of *Ae. albopictus* and *Ae. aegypti* in Yaoundé; (**a**) House index (HI); (**b**) Breteau index (BI); and (**c**) container index (CI). Black and green dashed lines represent the thresholds of dengue and yellow fever transmission respectively defined by Pan American Health Organization and WHO.

The house (HI), Breteau (BI), and container indices (CI) were assessed to establish the potential risk of dengue and yellow fever transmission in Yaoundé. The overall HI was 40.5% (95%CI, 38.2–42.8) in Yaoundé and varied from 23 in Yaoundé II to 45% in Yaoundé III with no significant difference (*χ*^2^ = 10.2, *df* = 6, *p* > 0.05) (Table S2). Analysis conducted between species revealed that the overall HI for *Ae. albopictus* (38.3%; 95%CI, 36.0–40.6%) was significantly higher than the HI for *Ae. aegypti* (25.7%; 95%CI, 23.7–27.8%) (*χ*^2^ = 24.7, *df* = 6, *p* < 0.001). Analysis according to borough showed that the HIs for *Ae. albopictus* were significantly higher than those for *Ae. aegypti* (*p* < 0.05) (Table S2, Figure 1a). Globally, the BI for *Aedes* spp. was 80.5% (95%CI, 74.4–86.6%) in Yaoundé and ranged from 50% to 108% with no significant difference (ANOVA, F = 1.44, *df* = 6, *p* > 0.05). Analysis according to the *Aedes* species showed that the BI for *Ae. albopictus* 71.8% (95%CI, 66.2–77.4%) was significantly higher compared to that of *Ae. aegypti*, 40.9% (95%CI, 37–44.8%) (ANOVA, F = 320.1, *df* = 1, *p* < 0.001). However, the BI for *Ae. albopictus* populations varied from 45% in Yaoundé II to 92% in Yaoundé VII with no significant difference (ANOVA, F = 0.93, *df* = 6, *p* > 0.05). In contrast, BIs in *Ae. aegypti* populations varied significantly from 26% in Yaoundé V to 74% in Yaoundé VII (ANOVA, F = 3.20, *df* = 6, *p* < 0.01) (Table S2, Figure 1b). Similar analysis was performed for the container index and revealed that globally the CI was 33.2% (95%CI, 31.8–34.6%) with no significant difference according to borough (*χ*^2^ = 13.3, *df* = 6, *p* > 0.05). A similar trend to the HI and BI was observed for the CI when analysing according to *Aedes* species; the CI calculated for *Ae. albopictus* (29.6%; 95%CI, 28.2–31%) was significantly higher than that of *Ae. aegypti* (16.9%; 95%CI, 15.7–18%) in Yaoundé (*χ*^2^ = 38.4, *df* = 6, *p* < 0.001) (Table S2, Figure 1c). Based on the estimated indices for *Ae. aegypti* in reference to threshold levels for dengue and yellow fever transmissions established by Pan American Health Organization (PAHO) [43] and World Health Organization (WHO) [44], the city of Yaoundé could be classified as a high-potential risk area for arbovirus transmissions, notably dengue and yellow fever (Figure 1).

**Figure 2:**
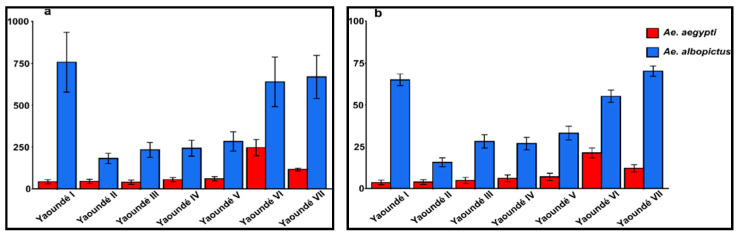
Pupae-based indices for *Ae. albopictus* and *Ae. aegypti* in Yaoundé; (**a**) pupae index (PI) and (**b**) pupae per person index (PPI).

Furthermore, the pupae index (PI) and the pupae per person index (PPI) were computed revealing an overall PI of 503.1% (95%CI, 461.4–544.9%) which varied significantly from 226.7% in Yaoundé II to 785.4% in Yaoundé VII (ANOVA, F = 8.9, *p* < 0.05). The pupae index for *Ae. albopictus* was significantly higher than that of *Ae. aegypti* (ANOVA, F = 9.05, *p* < 0.01) (Figure 2a). The mean PPI was 50.3% (95%CI, 48.8–51.8%) with a significant difference observed between PPIs for *Ae. albopictus* (44.6%, 95%CI, 43.2–48.1%) and *Ae. aegypti* (5.7%, 95%CI, 5.0–5.7%) (*p* < 0.001) (Figure 2b).

### 2.3. Container Prevalence and Preferences of Ae. albopictus and Ae. aegypti

**Figure 3:**
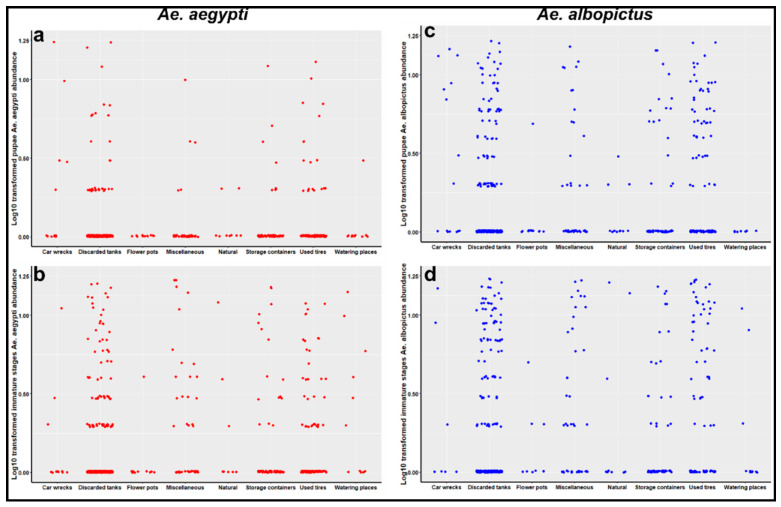
Total abundance of pupae and of pupae and larvae of *Ae. aegypti* (**a**,**b**) and *Ae. albopictus* (**c**,**d**), respectively, per type of container. Each dot represents the log10 transform of the abundance of containers infested by immature stages of *Ae. albopictus* and *Ae. aegypti*.

The various containers prospected were classified into three categories: domestic (watering places, flowerpots, and storage containers), peri-domestic (discarded tanks, used tyres, car wrecks, and miscellaneous), and natural (snail and coconut shells, tree and rock holes, and axils of plants). Peri-domestic containers were the most prevalent and the most infested category with a prevalence of infestation of 76.2% and 13.6% for *Ae. albopictus* and *Ae. aegypti* respectively (Table 2). The most productive containers for both species were discarded tanks and used tyres, although the distribution of immature stages and pupae were over-dispersed in all container types (Figure 3).

### 2.4. Environmental Characteristics Influencing the Presence of Aedes Species

The analysis revealed that the presence of immature stages of *Ae. aegypti* and *Ae. albopictus* was positively associated with the presence of plant debris inside the container and water with organic materials (*p* < 0.01). The presence of immature stages of *Ae. aegypti* and *Ae. albopictus* was also associated with flowerpots and used tyres, respectively (*p* < 0.05) (Table 3). The presence of *Ae. albopictus* pupae were also associated with containers of low capacity and containers made of rubber (*p* < 0.05), while the presence of *Ae. aegypti* pupae was associated with containers at a high distance from the ground (*p* < 0.05) (Table 3).

### 2.5. Spatial Distribution of Immature Stages of Ae. albopictus and Ae. aegypti

**Figure 4:**
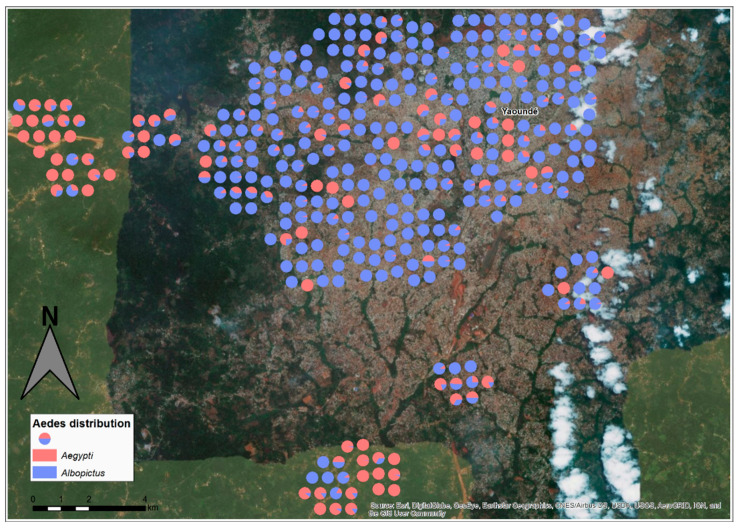
Spatial distribution of *Aedes albopictus* and *Aedes aegypti* in Yaoundé city. Each pie chart represents a positive container with the proportion of *Ae. albopictus* and *Ae. aegypti*. It is clearly observed that *Ae. albopictus* (in blue) is predominant in downtown and suburban areas whereas *Ae. aegypti* (in red) is predominant in rural areas, although it is present in some parts of the downtown areas.

A total of 10,801 specimens of *Ae. albopictus* and/or *Ae. aegypti* were identified after adults emerged, encompassing 84.95% *Ae. albopictus* and 15.05% *Ae. aegypti* (Table 2). The spatial distribution of the two species showed that both *Aedes* species were found across Yaoundé with a significant prevalence of *Ae. albopictus* in the suburb as well as downtown (*p* < 0.001), suggesting the efficient expansion and competitive advantage of this species. However, in the rural area no significant difference was found between the two *Aedes* species (*p* > 0.05), although *Ae. aegypti* seemed dominant. Indeed, *Ae. albopictus* was significantly more prevalent in almost all the neighbourhoods than *Ae. aegypti* (*p* < 0.05), except in Afanoyoa II and Eyang (two rural areas) where *Ae. aegypti* was significantly more prevalent (*p* < 0.001) (Table S1, Figure 4 and Figure S1).

## 3. Discussion

This study presented the current geographical distribution, the level of infestation, and the factors governing the presence of *Ae. albopictus* and *Ae. aegypti* in Yaoundé as well as the entomological risk for large arbovirus outbreaks based on *Stegomyia* indices. Our analysis confirmed the co-occurrence of both *Ae. aegypti* and *Ae. albopictus* across Yaoundé with a predominance of *Ae. albopictus* in downtown and suburban areas. This observation is in accordance with previous data collected in Yaoundé in 2007 highlighting the predominance of *Ae. albopictus* in this city [18]. A spot of *Ae. aegypti* persisting downtown matches the previous observations made in 2017 in the same city [13]. In fact, *Ae. albopictus* was first recorded in Cameroon in the early 2000s [11] and has rapidly colonized all human-domesticated environments in the southern part of the country [33,34]. The predominance of *Ae. albopictus* across Yaoundé confirms the competitive advantage of this species on the native species *Ae. aegypti* as was suspected previously [18]. These observations are consistent with other studies reported in several countries in the world in areas invaded by *Ae. albopictus* such as in Brazil [22,45], Florida [14,24], and Australia [26]. However, the mechanisms by which competition takes place are not well known, but some authors believe that it could happen at the pre-imaginal phase and that several factors such as temperature, precipitation, response to symbionts, predators, and chemical interferences that retard growth are the main drivers [25,46]. Also, other work has shown that the two species are able to mate in nature and that *Ae. albopictus* males effectively sterilize *Ae. aegypti* females [47,48,49]. The authors suggest that this form of mating interference, called satyrization, could explain the competitive displacement of resident *Ae. aegypti* by the invasive *Ae. albopictus* where they co-occur. Surprisingly, the coexistence of *Ae. aegypti* and *Ae. albopictus* was reported in Peninsular Florida (USA) two decades after competitive displacement, suggesting a resistance to mating interference [17]. The abundance of *Ae. aegypti* found in rural areas located in two different boroughs in Yaoundé could suggest a resistance to mating interference with *Ae. albopictus* among this population, which allows them to co-occur in this area, and further investigations are needed to elucidate. It is important to underline that this study was carried out during the raining season only, although it was demonstrated that seasonality can affect the pattern of abundance of *Ae. aegypti* and *Ae. albopictus* [19,50]. The variation in abundance between the two species would probably be due to the difference in the tolerance of desiccation of the eggs of both species [51]. However, previous data collected in Central Africa suggest that the variation in abundance between *Ae. aegypti* and *Ae. albopictus* depends on the difference of time between the rainy season and the dry season among locations [13]. Surprisingly, no immature stages were found in the Briquetrie (Muslim) neighbourhood in the houses surveyed; however, residents notified us of the use of larvicide to treat the potential breeding sites. Further studies including socio-anthropological aspects are needed to elucidate further.

Overall, the immature stages of both *Ae. aegypti* and *Ae. albopictus* preferentially colonized peri-domestic containers, particularly discarded tanks and used tyres. These observations are consistent with previous results reported in Central Africa [13,19]. However, the opposite situation was found in other parts of the world, particularly in Asia, where domestic containers such as water storage tanks represent the bulk of infested containers for *Ae. aegypti* [52]. Interestingly, it has been clearly established that in major unplanned urban cities, improved waste management through the physical removal of used containers reduces the quantity of mosquito breeding sites and thus decreases *Aedes* densities [1,53,54].

Both species breed in the same type of container, notably flowerpots, discarded tanks, used tyres, and car wrecks filled with turbid water, and are associated with plant debris inside. These outcomes highlight the impact of micro-environmental factors on the presence of *Aedes* spp. inside breeding sites. In fact, the presence of organic matters inside the larval habitats could serve as food resources [19,55] or a micro-habitat to hide and avoid predators [55,56]. The sympatric situations found in certain containers suggest possible competition for resources and other ecological interactions in the larval stage which may influence physiological characteristics like body size and wing length, and thus affect adult vector competence for arboviruses [54,57].

Overall, the infestation indices were higher compared to the thresholds established by WHO for dengue virus (DENV) [43] and yellow fever virus (YFV) [44] transmission. In fact, estimated risk values suggest that Yaoundé is at high potential risk of dengue outbreaks for both species and a high potential risk of yellow fever outbreaks for *Ae. albopictus*. These results highlight a higher potential of human exposure to the bites of *Ae. aegypti* and/or *Ae. albopictus* females in Yaoundé depending to the borough or neighbourhood. Indeed, previous studies based on *Stegomyia* indices have shown that high indices coincided with dengue outbreaks in some African countries such as Kenya [58], Ethiopia [59], and Tanzania [60]. The higher indices for *Ae. albopictus* compared to those of *Ae. aegypti* previously recorded in 2007 in Yaoundé [18] show a stability of the potential risk in this city. Such potential risk is also similar to that observed in other central African cities such as Bangui in the Central African Republic [19]. Interestingly, it was recently demonstrated that *Ae. aegypti* and *Ae. albopictus* populations from Yaoundé are able to transmit DENV [41] and Zika virus (ZIKV) [42], further increasing such potential risk. The same was observed of the *Ae. aegypti* population for YFV [40]. Additional studies including a dynamic of abundance of each species depending on the season and trophic behaviour of each species are required to establish the epidemiological importance of each species.

## 4. Materials and Methods

### 4.1. Ethics Statement

Ethical clearance N°2017/05/911/CE/CNERSH/SP was delivered by the Cameroonian National Ethics Committee for Human Health Research for this study. An oral consent form was obtained from the head or representative of each household prior to the survey.

### 4.2. Study Sites

**Figure 5:**
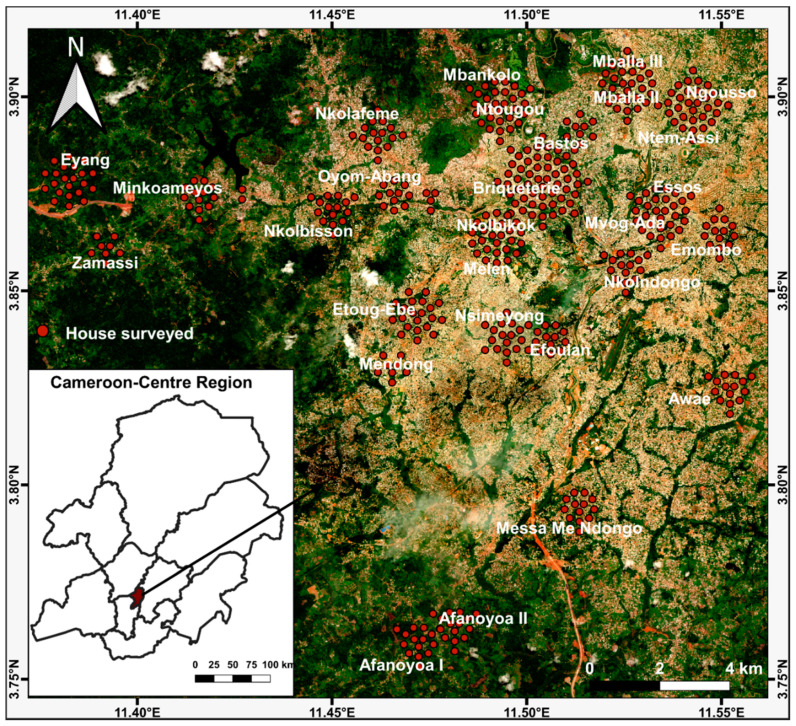
Geographical distribution of prospected houses in Yaoundé. Immature stages were sampled in cluster of 15 houses in each neighbourhood.

The study was carried out in Yaoundé (03°51′ N, 11°30′ E), the capital city of Cameroon, one of the most urbanized cities in the country with around 15,900 ha of urbanized areas divided into seven boroughs [61] (Figure 5). The city is 800 m above sea level and the environment is characterized by gentle rolling chains of hills, numerous valleys, and wetlands, and the remnants of the forest around these areas are being rapidly destroyed. The climate is sub-equatorial Guinean (mean annual rainfall and temperature of 1600 mm and 25 °C, respectively) with two distinct rainy seasons, the first extending from March to June and the second from September to November [62]. The agglomeration consists of more administrative and commercial structures discreetly distributed throughout the city [63]. From 2000 to 2014, the population has substantially doubled, reaching 2.6 million. This rapid growth has brought about an increase in the need for space and settlement which are reflected by the growing extension of houses in the suburbs of the city, peripheral neighbourhoods being mostly populated by waves of recent arrivals [61,63].

### 4.3. Study Design

A cross-sectional survey was carried out in Yaoundé, in April–May 2018, corresponding to the rainy season. Entomological surveys were undertaken in clusters of houses sampled randomly, each cluster consisting of 15 houses per neighbourhood in each of the seven boroughs (Figure 1). Each selected house was geo-referenced with a Global Positioning System (GPS, Garmin eTrex^®^) and inspected to record all natural and artificial water-holding containers (potential containers) and those containing at least one immature stage (larvae or pupae) of *Aedes* (positive containers). Positive and potential containers were geo-referenced, and the nature, mobility, material, and colour of the container, the distance between the container and the nearest building, the nearest plant and the ground, the container volume, the volume, source (rain, tap water, drilling water), and quality (clear, tinted) of water, the presence of plant debris inside the container, the presence of vegetation around the container, sun exposure, and the number of inhabitants per house were recorded. Potential containers were classified into three categories, domestic, peri-domestic, and natural based on the source and the use of the water. Domestic containers (e.g., storage containers) were defined as human-filled receptacles, whereas peri-domestic (e.g., used tyres and discarded tanks) and natural (e.g., tree and rock holes) containers were those filled by rain or humans. Whenever they were present, larvae and/or pupae were collected and returned to the insectarium at the Centre for Research in Infectious Diseases and isolated from predators such as *Culex tigripes* and *Toxorynchites* sp. larvae. For each container, pupae were isolated from larvae, counted, and reared to adults. Larvae were also reared to adults. The adults that emerged were identified using morphological identification keys [64,65]. The number of immature stages of *Ae. albopictus* and *Ae. aegypti* was estimated from the proportion of emerging adults of each species.

### 4.4. Entomological Indices

The level of infestation of *Ae. aegypti* and *Ae. albopictus* including the house index (HI, percentage of houses infested with larvae and/or pupae), the Breteau index (BI, number of positive containers per 100 houses inspected), and the container index (CI, percentage of water-holding containers infested with larvae and/or pupae) was assessed. Estimated thresholds of HI, BI, and CI references have been established by WHO for dengue and yellow fever transmission. Whenever HI > 35%, BI > 50%, and CI > 20%, the city is considered at high potential risk of the urban transmission of YFV, whereas HI < 4%, BI < 5%, and CI < 3% indicate that the city is considered at low potential risk of disease transmission [44]. Similarly, low HI (<0.1%), medium HI (0.1–5%), and high HI (>5%) were established for potential dengue transmission [43]. Additional indices based on the presence or absence and number of pupae were assessed, including the pupal index (PI, number of pupae per 100 houses) and the pupal per person index (PPI, number of pupae per 100 persons). To evaluate the most productive container, we assessed the productivity index which is defined as the percentage of pupae per container type among the prospected containers [52,55].

### 4.5. Data Analysis

Categorical and numerical variables were expressed as proportions and means respectively. Different proportions and means were compared using exact binomial and ANOVA tests respectively. Several environmental characteristics were recorded and the distribution of each variable was observed. Type of container (eight categories), type of container material (five categories), colour of material (three categories), mobility of the container (three categories), sun exposure (three categories), quality of water inside the container (three categories), plant debris inside the container (two categories), and presence of immature stages were defined as categorical variables, and the number of inhabitants, distance to the nearest building, plant, and ground, volume of the container, and the number of immature stages were defined as numerical variables. The level of association between environmental characteristics and the presence of larvae and/or pupae of *Ae. albopictus* and *Ae. aegypti* was assessed using a binary logistic regression from the generalized linear model (GLM) function. All statistical analyses were performed with R version 3.5.2 and RStudio version 1.1.463 [66], and *p*-value < 0.05 was considered statistically significant. The GPS coordinates of the neighbourhoods, prospected houses and positive containers of each species were projected onto maps with the open-source software QGIS [67].

## 5. Conclusions

This study highlights the predominance of *Ae. albopictus* over *Ae. aegypti* and emphasizes the existing risk of an arbovirus epidemic in Yaoundé. The results could help influence policies and contribute to the establishment of an arbovirus control program in Cameroon which is currently lacking.

## Figures and Tables

**Table 1:** Type of containers associated with the infestation of immature stages of *Ae. aegypti* and *Ae. albopictus* in Yaoundé.

		Type of Containers								
		Domestic			Peri-Domestic				Natural **	Total
Boroughs		Watering Place	Flowerpots	Storage Containers	Used Tyres	Discarded Tanks	Car Wrecks	Miscellaneous *		
**Yaoundé I**	**N**	3	4	15	23	52	2	12	1	112
	**% Positive**	33.3	25	53.3	47.8	38.5	50	58.3	0	4.4
	**% *Ae. albopictus* only**	0	100	37.5	72.7	55	100	85.7	0	61.2
	**% *Ae. aegypti* only**	0	0	0	9.1	5	0	0	0	4.1
	**% Mixed**	100	0	62.5	18.2	40	0	14.3	0	34.7
**Yaoundé II**	**N**	0	9	24	21	34	3	6		97
	**% Positive**	0	0	12.5	66.7	23.5	33.3	66.7		3.1
	**% *Ae. albopictus* only**	0	0	0	42.9	62.5	0	25	0	40
	**% *Ae. aegypti* only**	0	0	0	7.1	0	0	0	0	3.3
	**% Mixed**	0	0	100	50	37.5	100	75	0	56.7
**Yaoundé III**	**N**	1	1	25	13	119	2	9	7	177
	**% Positive**	0	100	28	30.8	25.2	100	44.4	28.6	2.8
	**% *Ae. albopictus* only**	0	100	57.1	75	50	50	25	50	52
	**% *Ae. aegypti* only**	0	0	14.3	25	10	50	25	0	14
	**% Mixed**	0	0	28.6	0	40	0	50	50	34
**Yaoundé IV**	**N**	0	4	6	14	59	1	6	0	90
	**% Positive**	0	50	33.3	57.1	32.2	0	83.3	0	4
	**% *Ae. albopictus* only**	0	100	50	25	42.1	0	40	0	41.7
	**% *Ae. aegypti* only**	0	0	0	0	10.5	0	0	0	5.6
	**% Mixed**	0	0	50	75	47.4	0	60	0	52.8
**Yaoundé V**	**N**	5	1	19	11	45	9	7	3	100
	**% Positive**	60	100	47.4	54.6	44.4	44.4	42.9	66.7	4.8
	**% *Ae. albopictus* only**	0	100	66.7	50	60	50	0	50	52.1
	**% *Ae. aegypti* only**	66.7	0	0	0	15	0	0	50	12.5
	**% Mixed**	33.3	0	33.3	50	25	50	100	0	35.4
**Yaoundé VI**	**N**	0	2	3	27	77	3	20	3	135
	**% Positive**	0	0	0	55.6	31.2	0	60	0	3.8
	**% *Ae. albopictus* only**	0	0	0	86.7	70.8	0	66.7	0	74.5
	**% *Ae. aegypti* only**	0	0	0	6.7	12.5	0	16.7	0	11.8
	**% Mixed**	0	0	0	6.7	16.7	0	16.7	0	13.7
**Yaoundé VII**	**N**	8	5	30	62	119	3	15	2	244
	**% Positive**	25	40	10	48.4	37	100	66.7	100	3.9
	**% *Ae. albopictus* only**	0	50	33.3	56.7	15.9	33.33	30	50	32.3
	**% *Ae. aegypti* only**	0	0	0	0	25	0	30	50	15.6
	**% Mixed**	100	50	66.7	43.3	59.1	66.67	40	0	52.1
**Total**	**N**	**17**	**26**	**122**	**171**	**505**	**23**	**75**	**16**	**955**
	**% Positive**	**35.3**	**26.9**	**26.2**	**51.5**	**32.7**	**47.83**	**60**	**37.5**	**37.7**
	**% *Ae. albopictus* only**	**0**	**85.7**	**46.9**	**59.1**	**45.5**	**45.45**	**46.7**	**50**	**49.2**
	**% *Ae. aegypti* only**	**33.3**	**0**	**3.1**	**4.6**	**13.9**	**9.1**	**13.33**	**33.3**	**10.8**
	**% Mixed**	**66.7**	**14.3**	**50**	**36.4**	**40.6**	**45.45**	**40**	**16.67**	**40**

N, total number of potential containers prospected; % Positive, percentage of containers infested with immature stages of *Ae. albopictus* and/or *Ae. aegypti*; % *Ae. albopictus* only, percentage of containers infested only with immature stages of *Ae. albopictus*; % *Ae. aegypti* only, percentage of containers infested only with immature stages of *Ae. aegypti*; % Mixed, percentage of containers infested with immature stages of *Ae. aegypti* and *Ae. albopictus*; *, Miscellaneous included mostly block holes, ground tarpaulin, roof gutters, motorcycle helmets and wrecks, debris of fridges and televisions; **, Natural included snail and coconut shells, tree and rock holes, and axils of plants.

**Table 2:** Level of infestation of *Ae. albopictus* and *Ae. aegypti* in different types of containers in Yaoundé.

	Type of Containers								
	Domestic			Peri-Domestic				Natural **	Total *n* (%)
Species	Watering Places *n* (%)	Flowerpots *n* (%)	Storage Containers *n* (%)	Used Tyres *n* (%)	Discarded Tanks *n* (%)	Car Wrecks *n* (%)	Miscellaneous * n (%)	Natural *n* (%)	
***Ae. albopictus***	42 (0.4)	176 (1.6)	654 (6.1)	2763 (25.6)	4008 (37.1)	482 (4.5)	982 (9.1)	68 (0.6)	9175 (85)
***Ae. aegypti***	33 (0.3)	3 (0.03)	105 (1)	214 (2)	930 (8.6)	105 (1)	221 (2.1)	15 (0.2)	1626 (15)
**Total**	75 (0.7)	179 (1.7)	759 (7)	2977 (27.6)	4938 (45.7)	587(5.4)	1203 (11.1)	83 (0.8)	10,801 (100)

*n*, abundance of immature stages; %, percentage of immature stages computed from the total number of immature stages collected; *, Miscellaneous included mostly block holes, ground tarpaulin, roof gutters, motorcycle helmets and wrecks, pot covers, other types of wrecks like fridges and televisions; **, Natural included snail and coconut shells, tree and rock holes, and axils of plants.

**Table 3:** Environmental characteristics of containers associated with the presence of pupae and larvae and/or pupae of *Ae. albopictus* and *Ae. aegypti*.

		Pupae					Larvae and Pupae				
			*Ae. aegypti*		*Ae. albopictus*			*Ae. aegypti*		*Ae. albopictus*	
Categories		Number	%	OR (CI 95%)	%	OR (CI 95%)	Number	%	OR (CI 95%)	%	OR (CI 95%)
**Distance to the nearest building (m)**	medium [5,6,7,8,9,10,11,12,13,14,15,16,17,18,19,20]	70	27.8	Ref	29.9	Ref	133	33.3	Ref	31	Ref
	low (<5)	163	66.7	1 (0.5–2.3)	69.2	1 (0.7–1.4)	272	60.6	0.8 (0.5–1.1)	66.9	0.9 (0.7–1.2)
	high (>20)	4	5.6	2.2 (0.3–8.8)	1	0.3 (0.1–1)	14	6.1	2.3 (0.9–5.3)	2.1	0.6 (0.2–1.5)
**Distance to the nearest plant (m)**	medium [5,6,7,8,9,10,11,12,13,14,15,16,17,18,19,20]	24	13.9	Ref	9.5	Ref	36	6.8	Ref	9.4	Ref
	low (<5)	211	86.1	0.7 (0.3–2.1)	89.6	1.1 (0.7–1.9)	381	93.2	1.6 (0.8–3.6)	89.9	1.1 (0.7–1.8)
	high (>20)	2		0.00 (NA–1.6 × 10^17^)	1	2.7 (0.3–17.4)	2		0 (NA–2.6 × 10^23^)	0.7	1.7 (0.2–10.8)
**Distance to the ground (m)**	medium [1,2,3]	21	5.6	Ref	9.5	Ref	42	10.6	Ref	9.8	Ref
	high [3,4,5]	2	2.8	21.5 (0.8–363.1) *	0.5	1.8 (0.1–20)	4	1.5	10.6 (1–236.8)	0.7	4.3 (0.4–94.5)
	low (<1)	214	91.7	1.7 (0.5–10.6)	90	1 (0.6–1.7)	373	87.9	0.8 (0.5–1.6)	89.5	0.9 (0.6–1.5)
**Sun exposure**	partially shaded	104	33.3	Ref	45.8	Ref	186	40.9	Ref	46	Ref
	exposed	94	47.2	2 (0.9–4.3)	38.3	1.2 (0.8–1.7)	170	44.7	1.6 (1.1–2.4) *	38.7	1.20 (0.9–1.6)
	shaded	39	19.4	1.8 (0.7–4.7)	15.9	1.1 (0.7–1.7)	63	14.4	1.1 (0.6–1.9)	15.3	1.04 (0.7–1.6)
**Material**	miscellaneous	12	2.8	Ref	5.5	Ref	25	5.3	Ref	6.3	Ref
	rubber	68	27.8	3.8 (0.7–69.6)	28.9	2.6 (1.2–5.1) *	101	16.7	1.2 (0.5–3)	27.5	2.1 (1.1–4) *
	metal	28	8.3	1.5 (0.2–29.9)	12.4	1.13 (0.5–2.6)	49	12.9	1.2 (0.5–3.3)	11.1	0.8 (0.4–1.6)
	natural	2		0 (0–3.2 × 10^11^)	1	1.3 (0.2–6.3)	2		0	0.7	0.7 (0.1–3.2)
	plastic	127	61.1	2.3 (0.5–42.1)	52.2	1 (0.51–2.05)	242	65.2	1.3 (0.6–3.3)	54.4	0.9 (0.5–1.6)
**Capacity of the container (L)**	high [20,21,22,23,24,25,26,27,28,29,30,31,32,33,34,35,36,37,38,39,40,41,42,43,44,45,46,47,48,49,50]	70	30.6	Ref	29.4	Ref	113	22	Ref	29.3	Ref
	medium [5,6,7,8,9,10,11,12,13,14,15,16,17,18,19,20]	82	38.9	0.86 (0.4–2)	33.8	0.7 (0.5–1.1)	138	29.5	0.9 (0.5–1.5)	34.5	0.7 (0.5–1)
	low <5	72	25	0.5 (0.2–1.3)	31.3	0.7 (0.4–1) *	144	42.4	1.4 (0.8–2.2)	30.7	0.6 (0.4–0.9) *
	very high > 50	13	5.6	0.6 (0.1–2.5)	5.5	0.6 (0.3–1.2)	24	6.1	1 (0.4–2.2)	5.6	0.6 (0.3–1.1)
**Volume of water (L)**	high [20,21,22,23,24,25,26,27,28,29,30,31,32,33,34,35,36,37,38,39,40,41,42,43,44,45,46,47,48,49,50]	5		Ref	2.5	Ref	9	1.5	Ref	2.4	Ref
	medium [5,6,7,8,9,10,11,12,13,14,15,16,17,18,19,20]	42	16.7	4 × 10^6^ (0–3.4 × 10^168^)	17.9	1.3 (0.5–4.2)	72	17.4	2.2 (0.6–14)	17.1	1.3 (0.6–3.5)
	low <5	188	83.3	4.9 × 10^6^ (0–4.1 × 10^166^)	78.9	1.5 (0.6–4.3)	335	80.3	2.5 (0.7–15.5)	79.8	1.6 (0.7–4)
	very high > 50	2		1 (0–8.7 × 10^15^)	1	0.5 (0.1–2.5)	3	0.8	0.7 (0–7.3)	0.7	0.3 (0.1–1.5)
**Origin of water**	well	1		Ref	0.5	Ref	2	0	Ref	0.7	Ref
	rain	227	97.2	6.1 × 10^5^ (0–NA)	95.5	1.1 (0.2–20.6)	407	98.5	942714.93 (0–NA)	96.5	0.6 (0.1–4.9)
	tap	9	2.8	6.5 × 10^5^ (0–9.5 × 10^164^)	4	1.9 (0.2–40)	10	1.5	5 × 10^5^ (486–9.1 × 1116)	2.8	0.7 (0.1–6.2)
	urine	0		1 (0–2.2 × 10^24^)		0 (NA–4.6 × 10^34^)	0		1 (0–1.7 × 10^7^)		0 (NA–3 × 10^41^)
**Quality of water**	clear	132	55.6	Ref	55.7	Ref	243	59.1	Ref	57.5	Ref
	polluted	6	2.8	0.3 (0.02–1.56)	2.5	0.3 (0.1–0.6) *	10	1.5	0.2 (0–0.5) *	2.8	0.3 (0.1–0.5) *
	turbid	99	41.7	2 (1–4) *	41.8	2.4 (1.8–3.4) *	166	39.4	1.9 (1.30–2.8) *	39.7	2.5 (1.8–3.4) *
**Presence of plant debris**	no	88	38.9	Ref	36.8	Ref	167	39.4	Ref	40.1	Ref
	yes	149	61.1	1.9 (1–3.9)	63.2	2.5 (1.8–3.5) *	252	60.6	2.1 (1.41–3) *	59.9	2.3 (1.8–3.1) *
**Type of container**	watering places	1	2.8	Ref		Ref	8	3.8	Ref	1	Ref
	miscellaneous	25	8.3	0.7 (0.1–14)	10.9	NA	51	14.4	0.8 (0.3–2.8)	11.1	3.5 (1–16)
	car wrecks	14	11.1	3.4 (0.4–69.5)	5	NA	14	3.8	0.7 (0.2–2.9)	3.1	3 (0.7–15.7)
	natural	3		0 (0–3.9 × 10^27^)	1.5	NA	6	1.5	0.3 (0–1.9)	1.4	1.6 (0.3–9.3)
	used tyres	69	27.8	1 (0.2–18.8)	29.4	NA	103	17.4	0.4 (0.1–1.3)	27.9	4.1 (1.3–18.3) *
	flowerpots	2		0 (0–1.4 × 10^20^)	1	NA	6	0.8	0.1 (0–0.7) *	1.7	1.1 (0.2–6.1)
	discarded tanks	101	38.9	0.46 (0.08–8.51)	43.3	NA	191	47.7	0.34 (0.12–1.10)	44.6	1.6 (0.5–7)
	storage containers	22	11.1	0.5 (0.1–11)	9	NA	40	10.6	0.3 (0.1–1.1)	9.1	1.3 (0.4–5.8)
**Colour**	mixed	31	2.8	Ref	14.9	Ref	69	19.7	Ref	15	Ref
	single	184	83.3	7.3 (1.6–131)	76.6	1.3 (0.8–2)	304	66.7	0.8 (0.5–1.3)	75.3	1.3 (0.9–1.9)
	transparent	22	13.9	6.6 (1.1–127.3)	8.5	0.7 (0.4–1.3)	46	13.6	0.9 (0.4–1.7)	9.8	0.8 (0.5–1.4)
**Mobility of the container**	fixed	6		Ref	3	Ref	8	1.5	Ref	2.1	Ref
	lightweight	222	94.4	1.6 × 10^6^ (0–NA)	93.5	0.6 (0.2–2.2)	395	94.7	0.9 (0.2–5.8)	94.1	0.5 (0.2–1.6)
	heavyweight	9	5.6	2.9 × 10^6^ (0–5.9 × 10^164^)	3.5	0.7 (0.2–3)	14	3.8	1.1 (0.2–8.2)	3.1	0.5 (0.1–1.9)

OR, odds ratio; CI, confidence interval; *, significant association; NA, not applicable; Ref, comparator group to estimate the OR.

## Supplementary Materials

The following are available online at https://www.mdpi.com/2076-0817/9/6/491/s1, Figure S1: Spatial distribution of abundance of immature stages of *Ae. albopictus* and *Ae. aegypti* per neighbourhood in Yaoundé. Table S1: Prevalence of immature stages of *Ae. albopictus* and *Ae. aegypti* and chi-squared test comparison of different neighbourhoods of Yaoundé. Table S2: Estimated dengue and yellow fever transmission potential risk levels based on house (HI), Breteau (BI), and container (CI) indices in Yaoundé.
