# Supplementary material for: Patterns of Ecological Adaptation of Aedes aegypti and Aedes albopictus and Stegomyia Indices Highlight the Potential Risk of Arbovirus Transmission in Yaoundé, the Capital City of Cameroon
Source: Pathogens. 2020 Jun 20;9(6):491. doi: 10.3390/pathogens9060491 (PMC7350347; doi:10.3390/pathogens9060491)
Supplement: Supplementary file 1 [file pathogens-09-00491-s001.pdf]

Table S1: Prevalence of immature stages of *Ae. albopictus* and *Ae. aegypti* and Chi-squared test comparison in different neighbourhoods of Yaoundé

		Ae. aegypti		*Ae. albopictus*			
**Boroughs**	**Neighbourhoods**	**N**	**%**	**N**	**%**	**df**	**Chi squared**
**Yaoundé I**	Bastos[a]	11	0.1	327	3	1	84.1*
	Mballa.II[a]	8	0.1	564	5.2	1	357.8*
	Mballa.III[a]	107	1	350	3.3	1	5.1*
	Ntougou[b]	4	0.1	351	3.3	1	278.7*
	**Total**	**130**	**1.2**	**1592**	**14.7**	**1**	**152.2***
**Yaoundé II**	Briqueterie[a]	0	0	0	0	NA	NA
	Mbankolo[b]	9	0.1	472	4.4	1	220.5*
	Messa[a]	127	1.2	56	0.5	1	0.8
	Tsinga[a]	80	0.7	224	2.1	1	2.4
	**Total**	**216**	**2**	**752**	**7**	**1**	**12.3***
**Yaoundé III**	Afanoyoa.I[c]	69	0.6	76	0.7	1	0.01
	Afanoyoa.II[c]	172	1.6	5	0.1	1	51.6*
	Efoulan[b]	11	0.1	404	3.7	1	130*
	Nsimeyong[b]	1	0.01	502	4.7	1	2324*
	**Total**	**253**	**2.3**	**987**	**9.1**	**1**	**19.7***
**Yaoundé IV**	Awae[b]	24	0.2	310	2.9	1	31.6*
	Emombo[a]	0	0	52	0.5	-	-
	Messa.Me.Ndongo[b]	72	0.7	145	1.3	1	0.7
	Nkolndongo[a]	21	0.2	348	3.2	1	47.1*
	**Total**	**117**	**1.1**	**855**	**7.9**	**1**	**43.1***
**Yaoundé V**	Essos[a]	55	0.5	337	3.1	1	13.4*
	Mvog.Ada[a]	97	0.9	174	1.6	1	0.6*
	Ngousso[b]	4	0.04	163	1.5	1	58.5*
	Ntem.Assi[a]	13	0.1	345	3.2	1	78.5*
	**Total**	**169**	**1.6**	**1019**	**9.4**	**1**	**39.6***
**Yaoundé VI**	Etoug.Ebe[b]	23	0.2	423	3.9	1	64.4*
	Melen[a]	1	0.01	184	1.7	1	310.1*
	Mendong[b]	5	0.1	150	1.4	1	38.9
	Nkolbikok[a]	1	0.01	536	5	1	2650*
	**Total**	**30**	**0.3**	**1293**	**12**	**1**	**492.3***
**Yaoundé VII**	Eyang[c]	289	2.7	30	0.3	1	20.7*
	Minkoameyos[c]	115	1.1	71	0.7	1	0.16
	Nkolafeme[c]	7	0.1	407	3.8	1	211.6*
	Nkolbisson[b]	98	0.9	882	8.2	1	58.1*
	Oyom.Abang[b]	84	0.8	1216	11.3	1	141.2*
	Zamassi[c]	118	1.1	71	0.66	1	0.17
	**Total**	**711**	**6.6**	**2677**	**23.9**	**1**	**50.3***
**Total**		**1626**	**15.1**	**9175**	**84.9**	**1**	**324.5***

	Type of neighbourhoods						
	**Downtown[a]**	54	15	101	28.1	1	11.3*
	**Suburban[b]**	67	18.6	161	44.7	1	36.6*
	**Rural[c]**	55	15.3	46	12.8	1	0.4
	**Total**	**176**	**48.9**	**308**	**85.6**	**1**	**27.5***

N, number of larvae and pupae collected; %, percentage; NA, not applicable; [a,b,c], neighbourhoods included in downtown, suburban and rural areas respectively; *, significant difference.

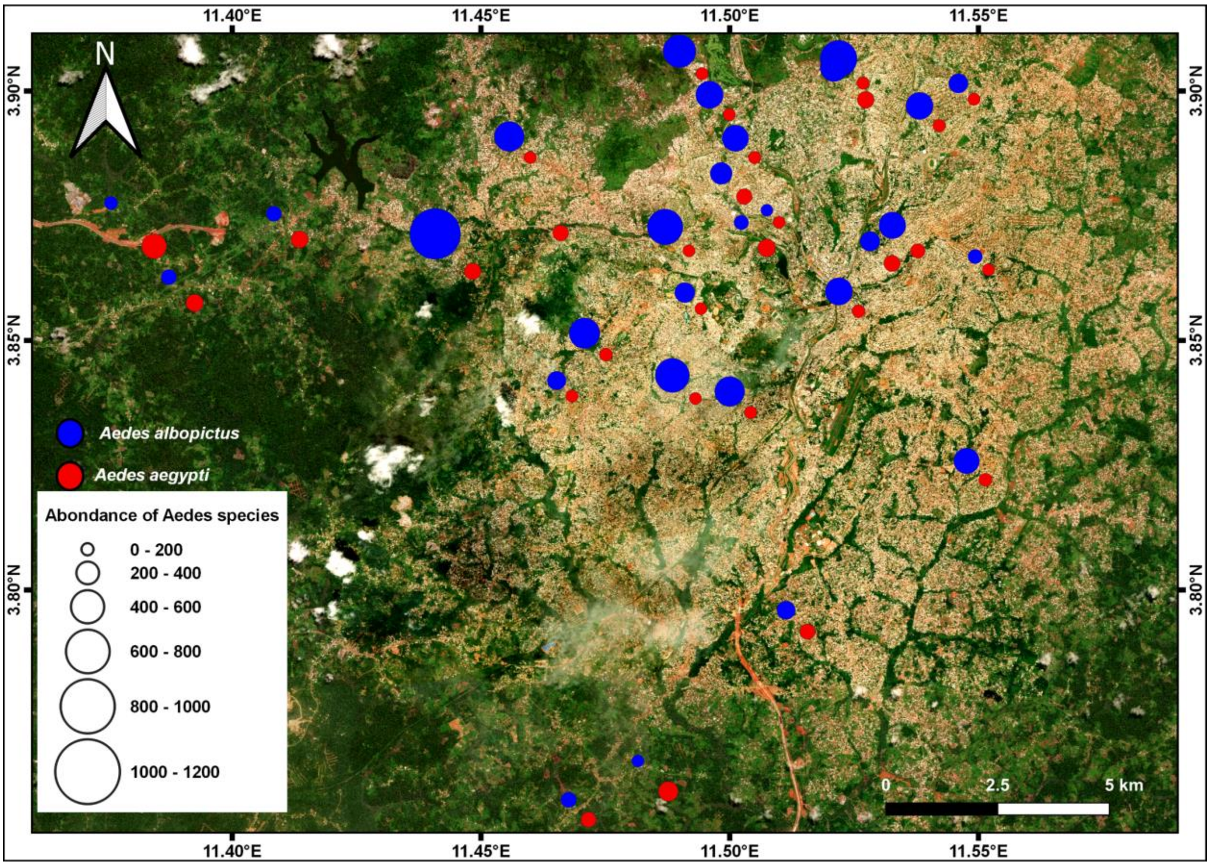

Figure S1. Spatial distribution of Abundance of immature stages of *Ae. albopictus* and *Ae. aegypti* per neighbourhood in Yaoundé.

Table S2: Estimated dengue and yellow fever transmissions risk levels based on House (HI), Breteau (BI), and Container (CI) indices in Yaoundé.

	House index-HI (95%CI)		Breteau index-BI (95%CI)		Container index-CI (95%CI)		Overall Indices (95%CI)			Risk level
**Boroughs**	***Ae. albopictus***	***Ae. aegypti***	***Ae. albopictus***	***Ae. aegypti***	***Ae. albopictus***	***Ae. aegypti***	**HI**	**BI**	**CI**	
**Yaoundé I**	45 (38.3-51.6)	23.3 (22-1.7)	78.3 (64.7-92)	31.7 (23.3-40.1)	35.6 (31.4-39.9)	14.4 (11.3-17.5)	45 (38.5-51.5)	81.7 (67.3-96)	37.1 (14.4-59.8)	**High**
**Yaoundé II**	23.3 (18.3-28.3)	16.7 (14.9-18.4)	48.3 (35.4-61.2)	30 (62.4-40.2)	23.5 (19.7-27.3)	14.4 (11.3-17.6)	23.3 (17.8-28.8)	50 (36.7-63.4)	24 (14.4-33.6)	**High**
**Yaoundé III**	42.2 (36.5-47.9)	37.8 (35.8-39.8)	60 (46.8-73.2)	51.1 (39.8-62.4)	23.1 (20-26.2)	12.9 (10.4-15.4)	48.9 (41.4-56.4)	83.33 (68.5-98.2)	26.88 (12.9-40.8)	**High**
**Yaoundé IV**	37 (33.6-40.4)	24.7 (23.6-25.7)	60.3 (48.3-72.3)	43.8 (32.6-55)	30 (25.8-34.4)	18.6 (14.9-22.3)	38.4 (32.6-44)	62 (46.9-77.2)	31.8 (18.6-45.1)	**High**
**Yaoundé V**	42.22 (37.4-47)	20 (19.2-20.8)	71.1 (56-86.2)	26.7 (17.5-35.9)	35 (30.6-39.4)	19.1 (15.6-22.8)	44.4 (37-52)	80 (65.4-94.6)	40 (19.2-60.8)	**High**
**Yaoundé VI**	37.3 (31.4-43.3)	14.7 (14.5-14.8)	81.3 (66-96.7)	18.67 (12.8-24.6)	30.4 (26.61-34.2)	8.8 (6.45-11.1)	41.33 (35.6-47)	85 (66.6-103.4)	34.5 (9.5-59.5)	**High**
**Yaoundé**	41.6	40.4	91	73	31.2	25	43.8	107.9	36.9	**High**

**VII**	(34.5-54.6)	(34.3-42.5)	(74.6-107.4)	(61.7-84.417)	(28.3-34)	(22.3-27.7)	(38.5-49.1)	(90.1-125.6)	(25-5.84)	
**Total**	**38.3**<br>**(36-40.6)**	**25.7**<br>**(23.7-27.8)**	**71.8**<br>**(66.2-77.4)**	**41**<br>**(37-44.8)**	**29.6**<br>**(28.2-31)**	**16.9**<br>**(15.7-18)**	**40.5**<br>**(38.2-42.8)**	**80.5**<br>**(74.4-86.6)**	**33.2**<br>**(31.8-34.6)**	**High**

95% CI; 95% Confidence Interval
